# Steroid Receptor Coactivator-2 Controls the Pentose Phosphate Pathway through RPIA in Human Endometrial Cancer Cells

**DOI:** 10.1038/s41598-018-31372-y

**Published:** 2018-09-03

**Authors:** Maria M. Szwarc, Ramakrishna Kommagani, Vasanta Putluri, Julien Dubrulle, Fabio Stossi, Michael A. Mancini, Cristian Coarfa, Rainer B. Lanz, Nagireddy Putluri, Francesco J. DeMayo, John P. Lydon, Bert W. O’Malley

**Affiliations:** 1 Department of Molecular & Cellular Biology, Baylor College of Medicine, Houston, Texas USA; 2 Department of Obstetrics & Gynecology, Washington University School of Medicine, St. Louis, Missouri USA; 3 Advanced Technology Cores, Dan L. Duncan Comprehensive Cancer Center, Baylor College of Medicine, Houston, Texas USA; 4 Reproductive and Developmental Biology Laboratory, National Institute of Environmental Health Sciences, Research Triangle Park, North Carolina, USA

## Abstract

Steroid receptor coactivator-2 (SRC-2) is a transcriptional coregulator that modulates the activity of many transcription factors. Levels of SRC-2 are elevated in endometrial biopsies from polycystic ovary syndrome patients, a population predisposed to endometrial cancer (EC). Increased expression of SRC-2 is also detected in neoplastic endometrium suggesting a causal link between elevated SRC-2 expression and the emergence of endometrial disorders that can lead to cancer. Here, we reveal that SRC-2 knockdown reduces EC cell proliferation and anchorage-independence. Additionally, SRC-2 is required to maintain cellular glycolytic capacity and oxidative phosphorylation, processes essential for EC cell proliferation. Importantly, SRC-2 is critical for the normal performance of the pentose phosphate pathway (PPP). Perturbation of the PPP due to loss of SRC-2 expression may result from the depletion of ribose-5-P isomerase (RPIA), a key enzyme of the PPP. As with SRC-2, RPIA knockdown reduces EC cell proliferation, which is accompanied by a decrease in glycolytic capacity and oxidative phosphorylation. Glucose metabolite tracking experiments confirmed that knockdown of SRC-2 and RPIA downregulates the metabolic rate of both glycolysis and the PPP, highlighting a novel regulatory cross-talk between glycolysis and the PPP modulated by SRC-2.

## Introduction

Endometrial cancer (EC) is currently the fourth most common cancer in women in the United States^1^. Since 2016, EC has been the sixth most deadly malignancy in the US^1^ which is a sharp increase in rank from eighth in 2013^2^. More alarming are predictions that show that EC incidence may double by 2030^3–5^, underscoring the urgency to improve diagnosis and treatment options for this gynecologic type of cancer. For EC patients, hysterectomy along with bilateral salpingo-oophorectomy is the primary treatment option, which can be combined with radiotherapy but with limited improvements in outcome^6–9^. In cases where fertility preservation is desired (14% of ECs occur in premenopausal women), the only targeted therapy used in management of EC is the use of progestins (medroxyprogesterone acetate or megestrol acetate), targeting the progesterone receptor (PGR)^10,11^.

The main transcriptional modulator of PGR activity in the healthy endometrium is steroid receptor coactivator-2 (SRC-2/NCOA2/TIF2/GRIP1) which has two closely related family members SRC-1 and SRC-3. Studies in human endometrial stromal cells (hESCs) *in vitro*^12^ and in a uterine-specific SRC-2 knockout mouse model^13^ revealed that SRC-2 is required for endometrial stromal cell (ESC) decidualization. Decidualization is a process of ESC proliferation and differentiation which is critical for successful embryo implantation and placentation. In hESCs, SRC-2 controls cell proliferation during decidualization through the regulation of glycolysis. Decidualizing cells require SRC-2 to maintain the expression of the enzyme 6-phosphofructo-2-kinase/fructose-2,6-biphosphatase 3 (PFKFB-3)^12^ which synthesizes fructose-2,6-bisphosphate, an allosteric activator of the glycolytic enzyme 6-phosphofructo-1-kinase (PFK-1)^14,15^.

In the case of endometrial pathology, SRC-2 is increased, along with SRC-3, in the endometrium of patients with polycystic ovary syndrome^16–18^, a population characterized by an increased EC risk. Additionally, these two coregulators are elevated in hyperplastic and neoplastic endometrium^19–21^. Together these descriptive findings suggest a causative link between the expression of SRC-2 and SRC-3 and the emergence of endometrial pathologies that may give rise to cancer. This hypothesis is supported by observations in an SRC-2 overexpressor mouse model^22^, in which endometrium-targeted increased levels of SRC-2 led to an impairment of decidualization associated with hyperproliferation of endometrial epithelial cells which are the primary cells of EC origin^23^. Upregulated expression of SRC-2 in the murine endometrium also led to increased sensitivity to estrogen exposure which is one of the main risk factors of endometrial cancer^24^.

In this study, we show that SRC-2 is indispensable for proliferation and anchorage-independence of human EC cells. The reduction of proliferation following SRC-2 knockdown is tightly linked to SRC-2′s role as a metabolic regulator. Measurements of metabolite levels and metabolic flux analysis revealed that SRC-2 is required for maintenance of glycolytic capacity and performance of the pentose phosphate pathway (PPP), due in part to its regulation of ribose 5-phosphate isomerase A (RPIA), an enzyme of the PPP.

## Results

### Proliferation and anchorage-independence of EC cells is maintained by SRC-2

**Figure 1:**
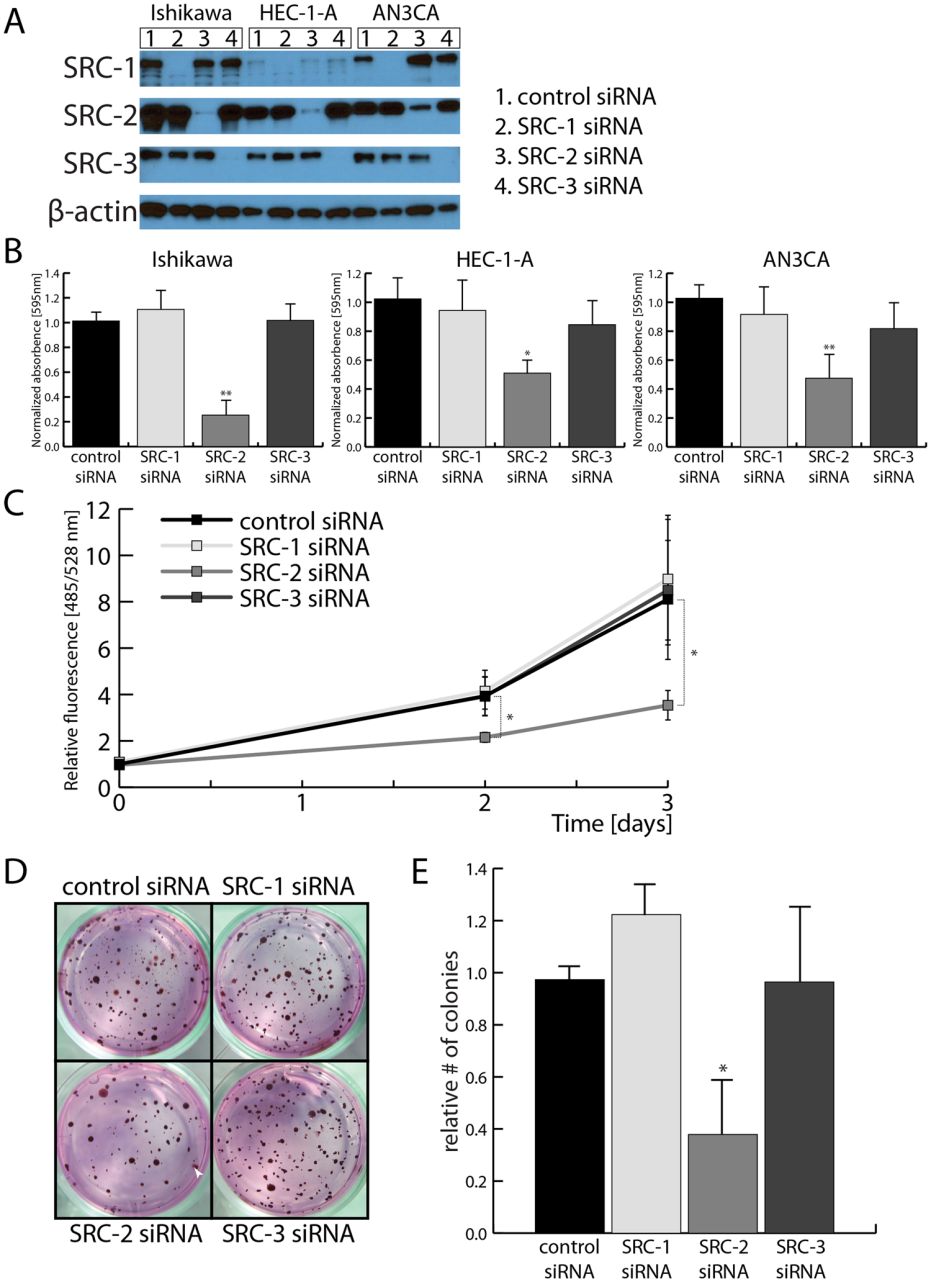
Maintenance of SRC-2 levels is required for EC cell accumulation and anchorage-independence. (**A**) Confirmation of siRNA-mediated knockdown of SRC-1, -2, and -3 by Western blot in EC cell lines. Uncropped images are provided in Supplemental Fig. S1. (**B**) Downregulation of SRC-2 but not -1 and -3 leads to reduction of cell colony formation by 3 EC cell lines (presented as quantification of extracted crystal violet used to stain formed colonies). (**C**) DNA content-based proliferation assay of Ishikawa cell line after knockdowns of SRCs demonstrates reduction of cell accumulation when SRC-2 levels are downregulated. (**D**) Examples of colonies formed by Ishikawa cells in soft agar after transfection with SRC-1, -2, or -3 siRNAs. A colony has been indicated with a white arrowhead. (**E**) Count of colonies formed in soft agar assay as shown in (**D**).

To study the role of SRCs in the maintenance of EC cell proliferative capacity, each of the three SRCs was knocked down using siRNAs in three EC cell lines: Ishikawa^25^ and HEC-1A^26^ – isolated from a well-differentiated EC tumors; and AN3CA^27^ – isolated from a EC metastasis (Fig. 1A, Supplemental Fig. S1). In a clonogenic cell survival assay, the survival and growth of EC cells plated at low density was tested. Reduction of SRC-2 levels, but not of SRC-1 or -3, led to a decrease in EC cell colony formation ability (Fig. 1B). Because the clonogenic cell survival method assays both cell proliferation and anchorage-independence, both of these properties were tested independently in the following experiments. A DNA content-based proliferation assay revealed that the accumulation of EC cells over 3 days was decreased when SRC-2, but not SRC-1 or SRC-3, levels were down-regulated (Fig. 1C). Testing the knockdown of SRCs in combination excluded the potential reciprocal compensation effects in the case of SRC-1 and SRC-3 knockdowns (Supplemental Fig. S2). As in the case of the proliferation assay, only SRC-2 knockdown caused a reduction of colony formation of cells plated in soft agar (Fig. 1D,E), demonstrating that SRC-2 is indispensable for both EC cell proliferation and anchorage-independence.

### SRC-2 is required to uphold glycolysis and generation of phosphopentoses in EC cells

**Figure 2:**
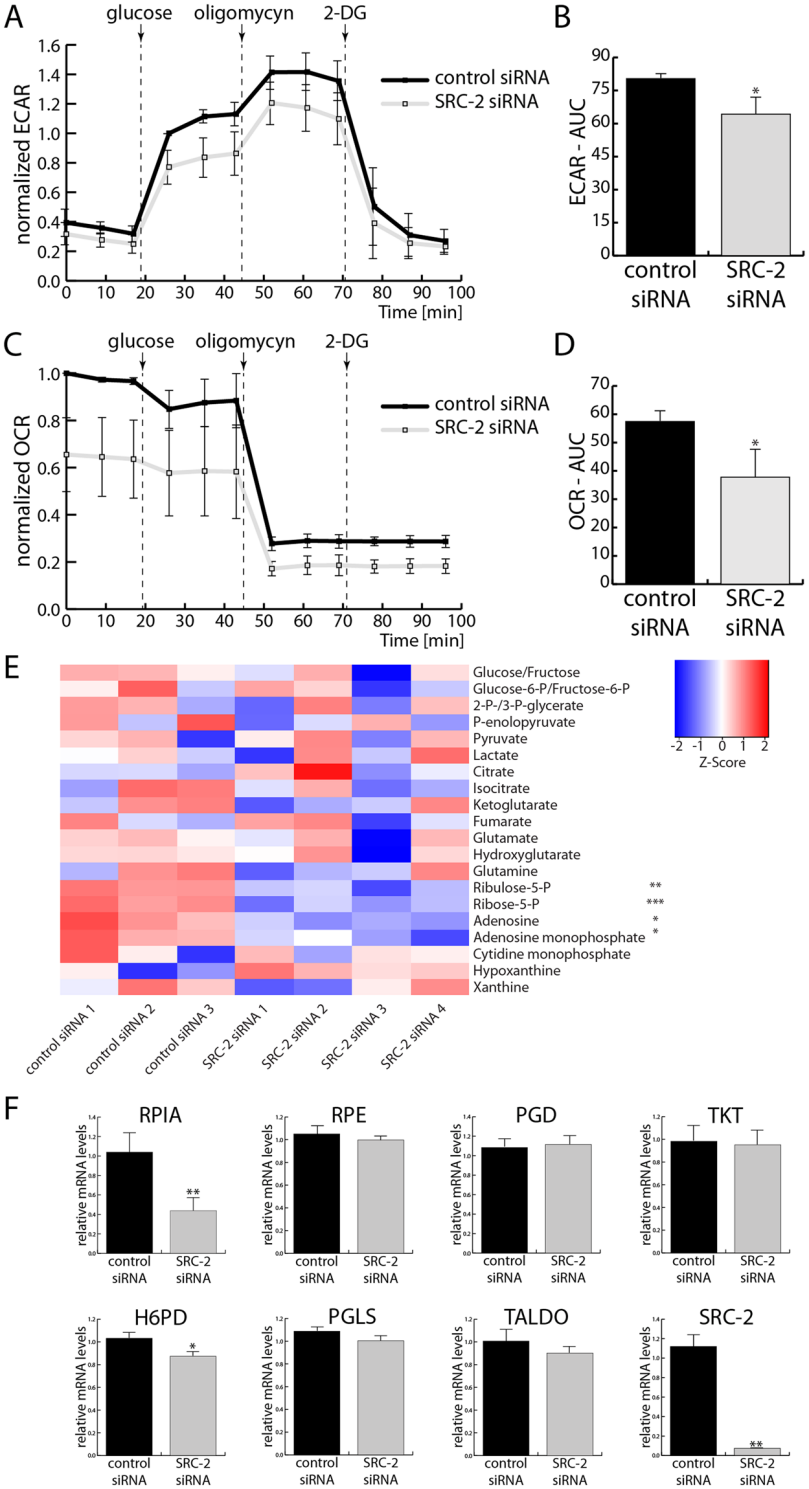
Upkeeping of glycolysis, OXPHOS, and the PPP in EC cell necessitates SRC-2. **(A)** Extracellular acidification rate (ECAR), measured before and after the addition of glucose and after the additions of oligomycin and 2-deoxyglucose (2-DG); quantification of the areas under the curves (AUC) are presented in **(B**). (**C)** Measurement of oxygen consumption rate (OCR) under the same conditions as described in (A) with AUC quantified in **(D**). (**E)** Quantification of steady metabolite levels in Ishikawa cells after control and SRC-2 knockdown. Each column represents a biological replicate of cells after either control or SRC-2 siRNA transfection (numbers 1–4 indicate biological replicates). Each row represents quantification of a metabolite which has been normalized with R heatmap.2() function’s default z-score row scaling. **(F)** Levels of mRNAs encoding enzymes of the PPP measured by RT-qPCR.

Because SRC-2 is crucial in maintaining the glycolytic capacity of decidualizing hESCs^12^, its role in EC cell metabolism was investigated. Lactate production (an output of glycolysis) which is measured by acidification of the medium was significantly decreased with SRC-2 knockdown (Fig. 2A,B). Specifically, both basal glycolytic capacity (measured before oligomycin treatment) and maximal glycolytic capacity (detected after oligomycin treatment) of EC cells were significantly reduced. Additionally, oxygen consumption, an output of oxidative phosphorylation (OXPHOS), was also significantly impaired following SRC-2 knockdown (Fig. 2C,D).

In hESCs, SRC-2 regulates glycolysis by upholding the expression of PFKFB3^12^ therefore the expression of PFKFB family members was tested first. Similarly as in the case of hESCs, SRC-2 knockdown in EC cells led to a moderate downregulation of PFKFB3 levels (Supplemental Fig. S3). In the case of other PFKFB family members, PFKFB1 transcripts were not detected by RT-qPCR, while no changes in PFKFB2 levels were found; PFKFB4 expression levels slightly increased with SRC-2 downregulation (Supplemental Fig. S3). Therefore due to the moderate opposing PFKFB3/4 expression changes, levels of metabolites of glycolysis, but also the tricarboxylic acid (TCA) cycle, and the pentose phosphate pathway (PPP) were assayed by liquid chromatography–mass spectrometry (LC-MS) (Fig. 2E). The only significant change in metabolite levels was the reduction of PPP metabolites with SRC-2 knockdown. In particular, SRC-2 knockdown led to the decrease in levels of phosphopentoses (ribulose-5-P and ribose-5-P) and their downstream metabolites (*i.e*. nucleotides and nucleosides). Based on this result, the expression levels of enzymes of the PPP were measured. Only one of them, ribose-5-phoshate isomerase (RPIA), was both significantly and markedly reduced with SRC-2 knockdown (Fig. 2F), suggesting a potential regulatory link between SRC-2 and the PPP in EC.

### Expression of RPIA is required for EC cell proliferation

**Figure 3:**
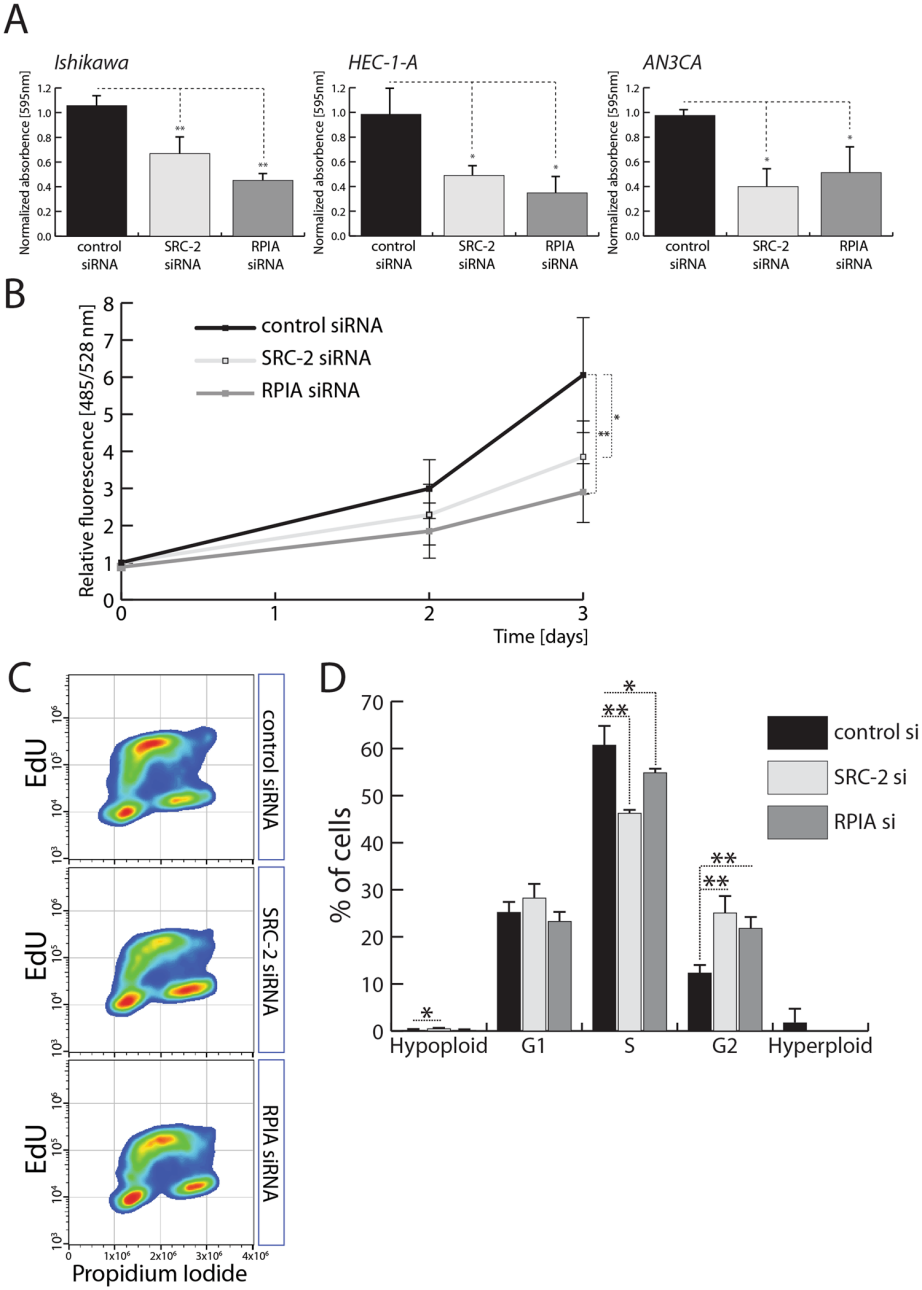
Expression of RPIA is required for EC cell proliferation. **(A)** Quantification of colony formation assays performed in EC cell lines – Ishikawa, HEC-1-A, and AN3CA – after control, SRC-2, and RPIA siRNA-mediated knockdown. **(B)** DNA content-based proliferation assay performed with Ishikawa cells after control, SRC-2, and RPIA downregulation. **(C)** Examples of scatterplots representing measurements of propidium iodide and EdU incorporation of Ishikawa cells transfected with control, SRC-2, and RPIA siRNAs. **(D)** Quantification of hypo- and hyperploid cell populations and cells in G1, S, and G2 phases of the cell cycle evaluated by quantification of PI and EdU intensity as presented in (**D**).

To determine whether the proliferative phenotype caused by reduction of SRC-2 levels is caused by the deregulation of RPIA, a clonogenic cell survival assay and a proliferation assay were performed in three EC cell lines. Knockdown of both SRC-2 and RPIA led to a significant downregulation of colony formation of the three EC cell lines (Fig. 3A). A DNA-content based proliferation assay in Ishikawa cells also confirmed these findings (Fig. 3B). Because reduction of SRC-2 levels resulted in a downregulation of nucleotide levels which could have led to a reduction of DNA synthesis and result in decreased cell proliferation, efficiency of DNA synthesis was evaluated. Both SRC-2 and RPIA knockdown caused a significant reduction in the population of cells in S-phase (Fig. 3C,D) as measured by levels of EdU incorporation over a period of 1 hour and by total DNA content, indicating a decrease of DNA synthesis. Interestingly, both SRC-2 and RPIA downregulation also caused an accumulation of cells in the G2 phase of the cell cycle, implying an impairment of EC cells entering into the mitotic phase of the cell cycle. On the other hand, synthesis of RNA was not affected by SRC-2 knockdown as measured by quantifying EU incorporation over 1 hour (Supplemental Fig. S4). Therefore, the effect of nucleotide depletion was primarily affecting new DNA, as opposed to RNA synthesis, and may in part explain the role of SRC-2 and RPIA in controlling EC cell proliferation. Additionally, reduction of SRC-2 and RPIA led to elevated activation of caspase-3 and caspase-7 indicating increased apoptosis (Supplemental Fig. S5).

### Glycolysis and PPP cross-talk regulated by SRC-2 and RPIA in EC cells

**Figure 4:**
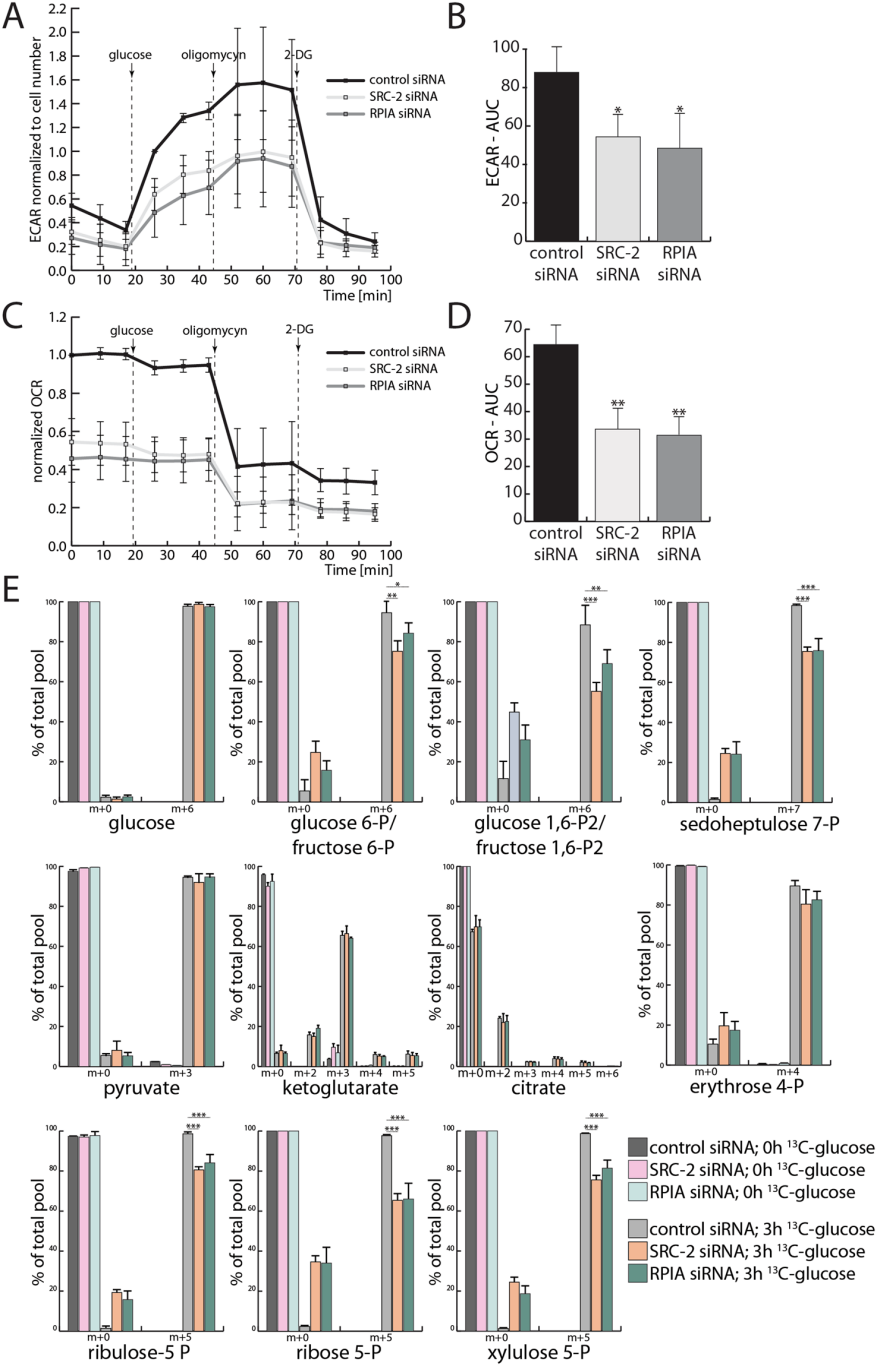
RPIA maintains glycolytic capacity, oxidative phosphorylation, and the PPP. **(A)** Extracellular acidification rate (ECAR) with quantification of the areas under the curves (AUC) presented in **(B)** of cells transfected with control, SRC-2, or RPIA siRNA. **(C)** Measurement of the oxygen consumption rate (OCR) under the same conditions as described in (**A**) with AUC quantified in **(D)**. **(E)** Proportion of ^12^C- and ^13^C-containing metabolites of glycolysis, the TCA cycle, and the PPP isolated from Ishikawa cells before and after a 3 h treatment with ^13^C(6)-glucose. The x axis labels indicate the metabolites containing only ^12^C (m + 0) or also ^13^C (m + 1, +2, +3, +4, +5, +6, and +7) isotopes.

When SRC-2 levels were decreased, a substantial downregulation of glycolytic capacity and OXPHOS occurred (Fig. 2A–D). However, only steady-state levels of PPP metabolites were affected by SRC-2 knockdown (Fig. 2E). Therefore, the regulation of these three metabolic pathways by SRC-2 and RPIA was investigated. As in the case of SRC-2 knockdown, downregulation of RPIA caused a reduction in both basal and maximal glycolytic capacity of EC cells (Fig. 4A,B) and in oxygen consumption (Fig. 4C,D). Assessment of expression levels of glycolytic enzymes revealed no dramatic changes when either SRC-2 or RPIA was knocked down (Supplemental Fig. S6). In fact, the glycolytic genes, whose expression was significantly altered by SRC-2 or RPIA knockdown, were upregulated. This finding may indicate an attempt of EC cells to compensate for the downregulation of glycolysis by SRC-2 or RPIA knockdown.

**Figure 5:**
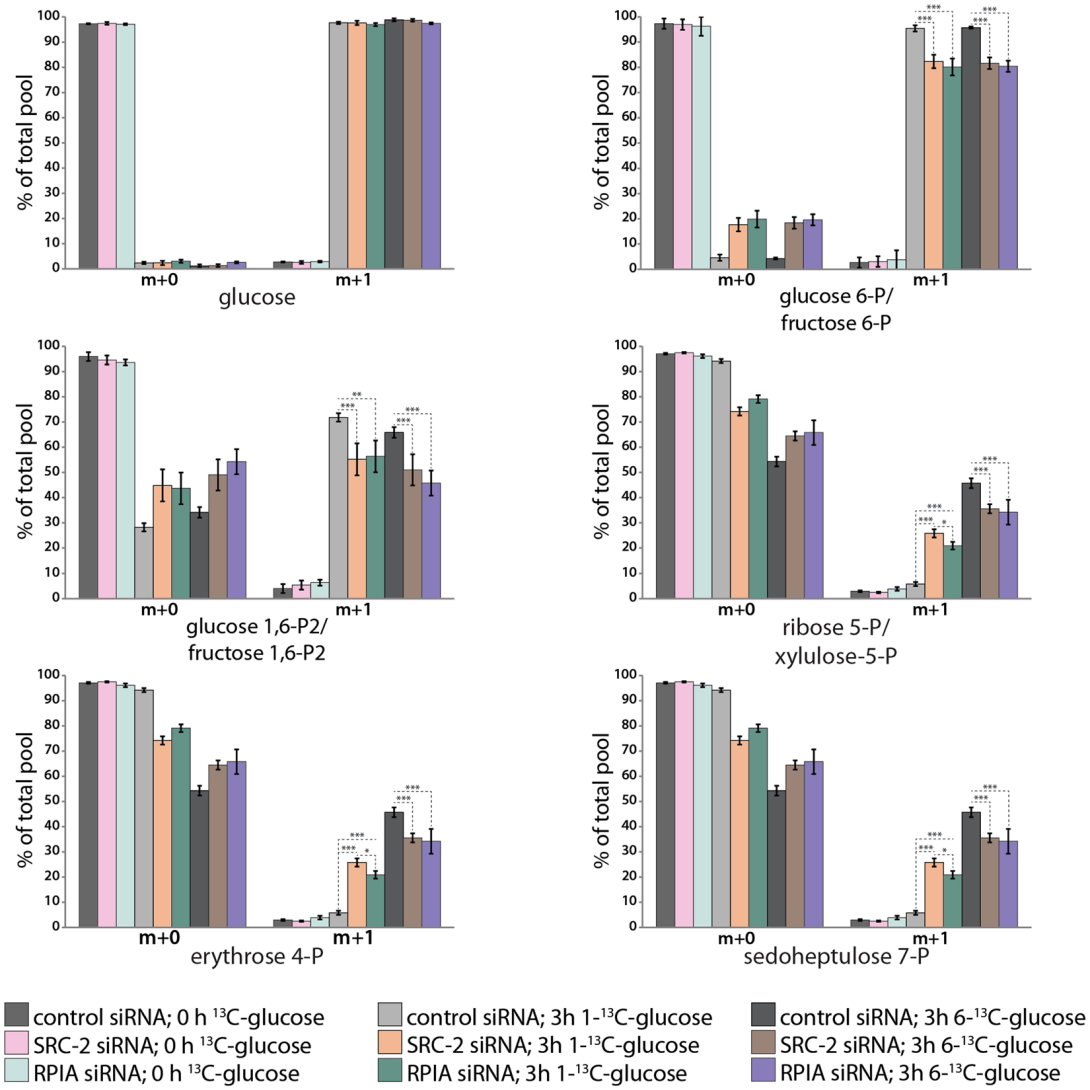
Glycolysis compensates PPP downregulation upon SRC-2/RPIA downregulation. Proportion of ^12^C- and ^13^C-containing metabolites of glycolysis and the PPP isolated from Ishikawa cells before and after 3 hours of treatment with 1-^13^C-glucose or 6-^13^C-glucose. The x axis labels indicate the metabolites containing only ^12^C (m + 0) or also ^13^C (m + 1) isotopes.

Therefore a direct approach to measure the synthesis of glycolytic and PPP metabolites with LC-MS was applied. Cells were treated with glucose which contained ^13^C in place of all its six carbons (^13^C(6)-glucose) and levels of newly generated ^13^C-containing glycolytic, TCA, and PPP metabolites were measured (Fig. 4E). Interestingly, the rate of generation of metabolites of the TCA cycle was not affected by SRC-2 or RPIA downregulation, indicating that the decrease in oxygen consumption was not a result of an impairment of the TCA cycle. However, as predicted by the glycolytic flux measurements (Fig. 4A,B) and PPP steady-level metabolite measurements (Fig. 2E), we found that knockdown of SRC-2 and RPIA led to the reduction in the generation of glycolytic metabolites and PPP phospho-sugars. Although the proportion of ^12^C to ^13^C pyruvate was not changed with SRC-2/RPIA knockdown, the overall levels of pyruvate have been found to be decreased (data not shown). Together, these findings demonstrate that SRC-2 and RPIA are required to uphold the flux of metabolites through both glycolysis and the PPP.

The PPP and glycolytic pathways first diverge at the level of glucose-6-P (G6P). However, both pathways generate overlapping metabolites further downstream: fructose-6-P (F6P) and glyceraldehyde-3-P (GA3P). Therefore the generation of phosphopentoses and other PPP-specific phospho-sugars may occur either through the canonical PPP pathway or through the utilization of glycolytic metabolites further downstream. To distinguish between the two pathways of PPP phospho-sugar generation, ^13^C tracking in cells treated with either 1-^13^C-glucose or 6-^13^C-glucose was performed (Fig. 5). Measurements of ^13^C-containing glycolytic metabolites generated from either 1-^13^C- or 6-^13^C-glucose revealed a downregulation of the glycolytic pathway when either SRC-2 or RPIA were knocked down, therefore confirming the findings of ^13^C tracking shown in Fig. 4E. Measurements of ^13^C-containing PPP phospho-sugars generated from 6-^13^C-glucose also confirmed the PPP impairment when SRC-2 or RPIA levels were downregulated. These measurements of ^13^C metabolites generated from 6-^13^C-glucose do not however distinguish between the generation of PPP phospho-sugars through the canonical PPP pathway (from G6P) or from F6P and GA3P. Cells treated with 1-^13^C-glucose will only generate ^13^C PPP phospho-sugars through metabolizing F6P and GA3P (Supplemental Fig. S7). Surprisingly, the levels of ^13^C PPP phospho-sugars generated from 1-^13^C-glucose were elevated when either SRC-2 or RPIA levels were reduced. This indicates that even though glycolysis and the PPP are overall impaired when SRC-2 or RPIA are knocked down, glycolysis still feeds back into the PPP and may even attempt to rescue diminished PPP phospho-sugar production.

## Discussion

The SRC family of transcriptional coregulators is implicated in the development of malignancies of multiple target tissues^28^. Due to their complex structure, SRCs are able to transactivate a broad spectrum of transcription factors and therefore are able to aid the survival and metastatic potential of cancer cells through a myriad of mechanisms^28,29^. In the case of the endometrium, overexpression of SRC-2 has been shown in a mouse model to induce increased proliferation of endometrial epithelial cells. These increased levels of SRC-2 also resulted in hypersensitivity to estrogen exposure unopposed by progesterone, a major EC risk factor^22^. Shortly after the generation of the SRC knockout mouse models, it became evident that SRCs are important regulators of carbohydrate, lipid, and amino acid metabolism in many different cell types^30^. However, the link between metabolism, cancer, and SRC biology is yet to be fully understood. This knowledge-gap is important to address due to the connection between metabolic syndrome and cancer^31–33^.

Metabolic syndrome is a cancer risk most highly associated with EC out of all cancer types^34^. In light of the increasing obesity epidemic^31^ and EC incidence^3–5^, the investigation of mechanistic connections between metabolism and EC progression is urgently required for the development of more effective prevention and treatment strategies for this type of cancer. In the healthy endometrium, SRC-2 has been found to maintain glycolysis through the regulation of PFKFB3^12^. In EC cells, we also discovered that SRC-2 drives glycolysis, although the mechanism through which it does so may in part differ. We found that SRC-2 upholds the expression of PFKFB3 in EC cells but negatively regulates PFKFB4. The upregulation of PFKFB4 in response to SRC-2 knockdown may compensate for downregulation of PFKFB3. However the enzymatic activity of PFKFB4 is not as clearly defined as that of PFKFB3^35–37^. Therefore, PFKFB4′s role in regulation of glycolysis in EC cells has to be further elucidated. Additionally, PFKFB4 has been recently shown to activate SRC-3 in breast cancer cells^38^ which may also be a potential compensation mechanism to SRC-2 downregulation in EC cells. Despite the opposing PFKFB3 and PFKFB4 expression changes in EC cells, we found that SRC-2 controls both glycolytic and PPP capacity. Whether PFKFB3 or PFKFB4 deregulation is causative for glycolytic or PPP impairment remains an open question. A more likely reason for this metabolic phenotype as shown by our work here is the downregulation of RPIA. Knockdown of SRC-2 caused a reduction in the levels of RPIA (but not other glycolytic or PPP enzymes) while decreasing RPIA levels phenocopied the metabolic effects of SRC-2 knockdown. Therefore, we propose that RPIA is the most likely mediator of SRC-2′s role in controlling glycolysis and the PPP. The exact mechanism of regulation of RPIA by SRC-2 has to be further investigated in the future. Available SRC-2 ChIP-sequencing datasets derived from mouse^39^ and human^40^ cells indicate direct binding of SRC-2 to the RPIA promoter and enhancer region, However an indirect regulation mechanism of RPIA should also be considered.

Another consequence of SRC-2 and RPIA downregulation is a reduction of EC cell proliferation which may have been directly caused by the impairment of the PPP. This is due to the tight connection between DNA replication and PPP activity^41^. The reduction of nucleotide content upon SRC-2/RPIA knockdown most likely directly blocked DNA replication, leading to a decrease in EC cells entering S phase of the cell cycle. This would be in agreement with findings by other groups, who showed that RPIA maintains proliferation of pancreatic adenocarcinoma, hepatocellular carcinoma, and colorectal adenocarcinoma cells^42–44^. Importantly, reduction of DNA synthesis was also previously noted when RPIA levels were downregulated^42^. In our studies, both SRC-2 and RPIA knockdown caused an accumulation of cells in G2 phase of the cell cycle indicating an impairment in mitosis. The defect in mitosis may also have been caused by reduction of nucleotide levels. Not only were the levels of nucleotides reduced but also their relative ratios (adenosine monophosphate levels were more reduced than cytidine monophosphate levels) were changed which has been previously linked to genomic instability^45^. This could be the underlying cause of cells being also blocked in G2 and not being able to pass the G2/M checkpoint.

An important question that arose from our findings is: how does reduction of RPIA cause a decrease in activity of both PPP and glycolysis? We expected that when the rate of either glycolysis or the PPP was reduced, it would have led to a shunt of metabolites into the other pathway and *vice versa*. Even more striking were our findings from ^13^C tracking in metabolites derived from 1-^13^C-glucose which showed that even though both glycolysis and the PPP were downregulated, glycolytic metabolites were still shunted into the PPP further downstream. These results indicate that SRC-2, acting through RPIA, is a positive regulator of glycolysis and PPP. And because the output of glycolysis/PPP was reduced already at the level of glucose-6-P, the positive regulation of these pathways by SRC-2/RPIA may occur as early as at the level of glucose phosphorylation.

We did not detect any notable reductions of glycolytic enzyme expression as a result of SRC-2/RPIA knockdown. Therefore, maintenance of glycolysis/PPP could be mediated post-transcriptionally through a metabolite of the SRC-2-RPIA axis. The concept of metabolic intermediates as regulators of metabolic pathways is known. For example, fructose-2,6-P2 can activate PFK-1 and drive glycolysis forward or direct the metabolites towards the PPP^14,15,46^. Similarly, 2-P-glycerate and 3-P-glycerate have been shown to stimulate the synthesis of serine and inhibit the PPP^47^. Another possibility through which the PPP-glycolysis cross-talk may be regulated is modulation of signaling proteins by metabolic intermediates as it has been found in the case of ribulose-5-P and AMP-dependent kinase (AMPK)^48^. The exact mechanism through which the SRC-2-RPIA axis simultaneously regulates the activity of glycolysis and the PPP will have to be further investigated. Due to SRC-2′s function as a coregulator of many transcription factors, other cellular and metabolic processes controlling EC cells proliferation and survival should also be investigated. This is of further importance in light of reduced oxygen consumption after SRC-2 knockdown indicating a global metabolic effect of SRC-2 in EC cells.

Together, our findings have uncovered a novel role for SRC-2 in the regulation of glycolysis and the PPP through RPIA, which in part explains regulation of EC cell proliferation by SRC-2. Because the SRC family fulfils a broad number of functions in the cell, the full spectrum of processes regulated by SRC-2 in EC cells has to be further evaluated. In light of the developing field of SRC small molecule inhibitors (SMI)^49–53^ and activators^54^, the targeting of SRC-2 in the uterus may be a promising avenue of EC prevention and treatment. The PPP has also been hypothesized and tested *in vivo* as a potential target for contraceptives delivered through intrauterine devices^55^. A similar application of these types of inhibitors could be investigated in models of increased risk of EC development.

## Material and Methods

### siRNA transfections

All cell culture media and supplements were purchased from ThermoFisher Scientific. Ishikawa cells were purchased from Sigma-Aldrich, HEC-1A and AN3CA cells were purchased from ATCC and maintained by Tissue Culture Core at Baylor College of Medicine. Due to increased number of experiments performed in Ishikawa cells, they were additionally confirmed by STR short tandem repeat (STR) analysis. Cells were plated into 6-well plates at 1–3 × 10^5^ cells/well and transfected with siRNAs using Lipofectamine RNAiMAX. Per transfection, 60 pmol of siRNA was complexed 6 μl of Lipofectamine. Cells were incubated with siRNA-transfection reagent complexes over a period of 48 h (two consecutive 24 h transfections). siRNAs for knockdown for each gene of interest were delivered as a mix of 4 siRNAs: 1) Non-targeting, Dharmacon (Lafayette, CO), D-001810-10-20; 2) SRC-2 – Dharmacon, L-020159-00-0005; and 3) RPIA – Qiagen (Valencia, CA), GS22934.

### Clonogenic cell survival assay

Following transfection, cells were plated into 12-well plates at 1000 cells per well and then grown for 8–10 days. The resulting colonies were stained with a 2.3% solution of crystal violet (Sigma-Aldrich, St. Louis, MO) for 15 min. To quantify colony formation, crystal violet was extracted with 10% acetic acid and its absorbance was measured at 590 nm.

### Cell proliferation assay

Following transfection, cells were plated into 96-well plates at 1000 cells per well. 3 hours later when cells have attached and every following 24 hours, cell culture medium was replaced with a DNA-binding fluorescent dye – Cyquant NF reagent (ThermoFisher Scientific, Waltham, MA) and incubated for 1 hour at 37 °C. Fluorescent signal was measured at 485 nm excitation and 528 nm emission.

### Soft agar assay

First, 12-well plates were filled with 0.5% agar in MEM with 10% FBS and Pen/Strep forming a bottom agar layer. Cells, following transfection, were plated into wells at 5000 cells per well in 0.35% agar in MEM with 10% FBS and Pen/Strep. Following agar solidification, agar was covered with MEM with 10% FBS and Pen/Strep and replaced every 4 days. After 28 days, formed cell colonies were stained with Cell Transformation Detection Assay Cell Stain Solution (Millipore, Billerica, MA) according to manufacturer’s instructions.

### Apoptosis assay

Following transfection, cells were plated into 96-well plates at 10000 cells per well. Apoptosis was quantified by measuring the activity of caspase-3 and caspase-7 activation using Caspase-Glo® 3/7 Assay (Promega, Madison, WI) according to manufacturer’s instructions. Alongside, cell number was quantified with Cyquant NF reagent.

### Glycolytic capacity and OXPHOS measurements

Following transfection, cells were plated into Seahorse XF24 Cell Culture Microplates at 5 × 10^4^ cells per well and allowed to attach overnight. Extracellular acidification rate and oxygen consumption rate were measured using a Seahorse XF Analyzer. Measurements were taken for each of the following conditions: (1) without glucose, (2) after addition of glucose (25 mM final concentration), (3) after addition of oligomycin (2.11 μM final concentration), and (4) after addition of 2-deoxyglucose (90 mM final concentration). Measurements were taken 3 times for each condition every 8.5 minutes. Parallel to the metabolic measurements, relative cell number was quantified with Cyquant NF reagent.

### Mass-spectrometric measurements of metabolite levels

After transfection, cells were trypsinized and washed two times with ice-cold DPBS. 3 × 10^6^ cells per sample were spun down and pellets were flash-frozen in a dry-ice ethanol bath and stored in dry ice. Metabolites were isolated and their levels were measured by liquid chromatography mass-spectrometry (LC-MS) by the Cancer Prevention Research Institute of Texas (CPRIT) Cancer Proteomics and Metabolomics Core at Baylor College of Medicine as described previously^56^.

### Measurements of ^13^C metabolites

Cells, following siRNA transfection, were either incubated in DMEM containing 25 mM ^12^C-glucose or treated for 3 hours with DMEM containing 25 mM ^13^C(6)-glucose; or with ^12^C-glucose, 1-^13^C-glucose, or 6-^13^C-glucose (Cambridge Isotope Laboratories, Inc., Tewksbury, MA). Samples were collected by flash-freezing cells with liquid nitrogen. Before cell freeze-down, reference medium from each sample was collected. Following the freeze-down, cells were scraped off and collected HPLC grade methanol:water/1:1. Metabolites were isolated and their levels were measured by LC-MS by the CPRIT Cancer Proteomics and Metabolomics Core at Baylor College of Medicine as described previously^56^.

### Cell cycle analysis

Cells were treated with 10 μM 5-ethynyl-2′-deoxyuridine (EdU) 1 hour prior to collection. Cells were fixed, permeabilized, and labeled for incorporated EdU with Click-iT® EdU Pacific Blue™ Flow Cytometry Assay Kit (ThermoFisher, Waltham, MA) according to manufacturer’s instructions. Following EdU labeling, cells were treated with RNase A (ThermoFisher, Waltham, MA) followed by 0.5 μM propidium iodide (ThemoFisher, Waltham, MA) for DNA labeling. Cells were analyzed at the Baylor College of Medicine Cell Cytometry and Cell Sorting Core using an Attune Flow Cytometer (ThermoFisher, Waltham, MA).

### *De novo* RNA synthesis measurements

Cells were treated with 1 mM 5-ethynyl uridine (EU) 1 hour prior cell fixation. The incorporated EU was labeled with Click-iT® RNA Alexa Fluor® 488 Imaging Kit (ThermoFisher, Waltham, MA) according to manufacturer’s instructions. Nuclei were stained with Hoechst 33342 (ThermoFisher, Waltham, MA) and cover glasses were mounted with ProLong Gold Antifade Mountant (ThermoFisher, Waltham, MA). Cells were imaged using GE Healthcare Inverted Deconvolution/Image Restoration Microscope at the Baylor College of Medicine Integrated Microscopy Core (IMC). Nuclear sizes and pixel intensities of nuclei and RNA synthesis were quantified performing custom scripts using Matlab software (MathWorks, Natick, MA) by the IMC.

### Western blot

Cells were lyzed with NP40 buffer containing cOmplete Mini protease inhibitors (Roche, Basel, Switzerland). Protein concentration was determined with Pierce™ BCA Protein Assay Kit (ThermoFisher, Waltham, MA). Protein (10 μg/lane) was resolved in a 4–15% gradient SDS-PAGE gel (Biorad Laboratories, Hercules, CA) before transferring to a polyvinylidene fluoride membrane (Millipore, Billerica, MA). Non-specific IgG binding was blocked with 5% milk in Tris-buffered saline (TBS) with 0.1% Tween. Immunoreactive bands were detected with the following antibodies: SRC-1 (1:1000 dilution, Bethyl Laboratories, Inc., Montgomery, TX), SRC-2 (1:2000 dilution, Bethyl Laboratories, Inc., Montgomery, TX), SRC-3 (1:1000 dilution, Santa Cruz Biotechnology, Santa Cruz, CA), and β-actin (loading control; 1:10000 dilution, Sigma-Aldrich, St. Louis, MO). The immunoreactive bands were detected with horseradish peroxidase-conjugated goat anti-rabbit or anti-mouse IgG (Santa Cruz Biotechnology, Santa Cruz, CA) and visualized with SuperSignal West Pico Chemiluminescent Substrate (Thermo Scientific, Rockford, IL).

### RNA isolation and reverse transcriptase quantitative PCR (RT-qPCR)

From cultured cells, total RNA was isolated using RNeasy Plus spin columns (Qiagen, Valencia, CA) according to manufacturer’s instructions. cDNA was prepared using SuperScript VILO cDNA Synthesis Kit (ThermoFisher, Waltham, MA) before RT-qPCR analysis was performed using TaqMan Universal Master Mix II with validated TaqMan Gene Expression Assays (ThermoFisher, Waltham, MA) listed in Supplemental Table ST1. Resultant amplicons were quantitated using a QuantStudio 12K Flex Real-Time PCR System; 18S rRNA was used as an endogenous control.

### Statistical analysis

Results are presented as averages ± standard deviations and are a summary of a minimum of three experiments performed on separate days, except for 1) the flow cytometry experiments in which due to the high sensitivity of propidium iodide staining intensity to cell dilution, a representative experiment was presented (one experiment out of three; each containing three biological replicates); and 2) the mass-spectrometric measurements of metabolites which included 3–5 biological replicates collected and processed on the same day because of electronics variation across days. Normality of data was evaluated by inspecting quantile comparison plots. Equality of variances was analyzed using the Bartlett test of homogeneity of variances. Statistical analyses were performed with either ANOVA or Kruskal-Wallis rank sum test with post hoc analysis performed with Student’s t-test, Tukey’s range test, or Wilcoxon rank-sum test in R Studio (R Studio Inc., Boston, MA). Multiple comparisons were adjusted with the Holm method. p ≥ 0.05 were considered as non-significant differences while *p < 0.05, **p ≤ 0.01, and ***p ≤ 0.001 were considered significant.

## Electronic supplementary material

Supplemental Files

## Data Availability

All data generated or analysed during this study are included in this published article (and its Supplementary Information files).
