# Supplementary material for: Steroid Receptor Coactivator-2 Controls the Pentose Phosphate Pathway through RPIA in Human Endometrial Cancer Cells
Source: Sci Rep. 2018 Sep 3;8:13134. doi: 10.1038/s41598-018-31372-y (PMC6120906; doi:10.1038/s41598-018-31372-y)
Supplement: Supplementary file 1 — Supplemental Files [file 41598_2018_31372_MOESM1_ESM.pdf]

SUPPLEMENTAL FIGURES AND TABLES

accompanying the manuscript:

Steroid Receptor Coactivator-2 Controls the Pentose Phosphate Pathway through RPIA in Human Endometrial Cancer Cells

Maria M. Szwarc[1], Ramakrishna Kommagani[2], Vasanta Putluri[1,3], Julien Dubrulle[1,3], Fabio Stossi[1,3], Michael A. Mancini[1,3], Cristian Coarfa[1], Rainer B. Lanz[1], Nagireddy Putluri[1,3], Francesco J. DeMayo[4], John P. Lydon[1**], and Bert W. O'Malley[1*]

[1]Department of Molecular & Cellular Biology, Baylor College of Medicine, Houston, Texas;
[2]Department of Obstetrics & Gynecology, Washington University School of Medicine, St. Louis, MO;
[3]Advanced Technology Cores, Dan L. Duncan Comprehensive Cancer Center, Baylor College of Medicine, Houston, Texas;
[4]Reproductive and Developmental Biology Laboratory, National Institute of Environmental Health Sciences, Research Triangle Park, North Carolina

Supplemental Figure S1. Uncropped Western blot images and additional exposures for Figure 1A

Supplemental Figure S2. Lack of compensation effect after SRC-1 or SRC-3 knockdown by other SRCs

DNA content-based proliferation assay performed on Ishikawa cells after double combinational knockdowns of SRCs measured over 3 days indicate that only SRC-2 downregulation causes a reduction in cell proliferation. No compensation by the other SRC underlies the lack of phenotype seen when SRC-1 and SRC-3 are downregulated.

Supplemental Figure S3. Expression of PFKFBs following SRC-2 knockdown

(A) RT-qPCR analysis of PFKFB3 and (B) PFKFB4 expression levels in Ishikawa cells after SRC-2 knockdown.

Supplemental Figure S4. No changes in RNA synthesis following SRC-2 and RPIA downregulation

Measurements of *de novo* RNA synthesis expressed as sum of pixel intensity per cell of fluorescently labeled incorporated EU.

Supplemental Figure S5. Increase in levels of apoptosis, measured in caspase-3 and caspase-7 activation, in Ishikawa cells with decreased SRC-2 or RPIA levels

Supplemental Figure S6. Reduction of glycolytic flux is not caused by downregulation of expression of glycolytic enzymes

RT-qPCR analysis of gene expression of key glycolytic enzymes

Supplemental Figure S7. Glycolysis and PPP cross-talk delineation with 1-^{13}C-glucose and 6-^{13}C-glucose tracking approach

Schematic representation of glycolysis and the PPP with ^{13}C marked as a red circle in cells treated with (A) 6-^{13}C-glucose or (B) 1-^{13}C-glucose.

Supplemental Table ST1. List of Taqman Gene Expression Assays

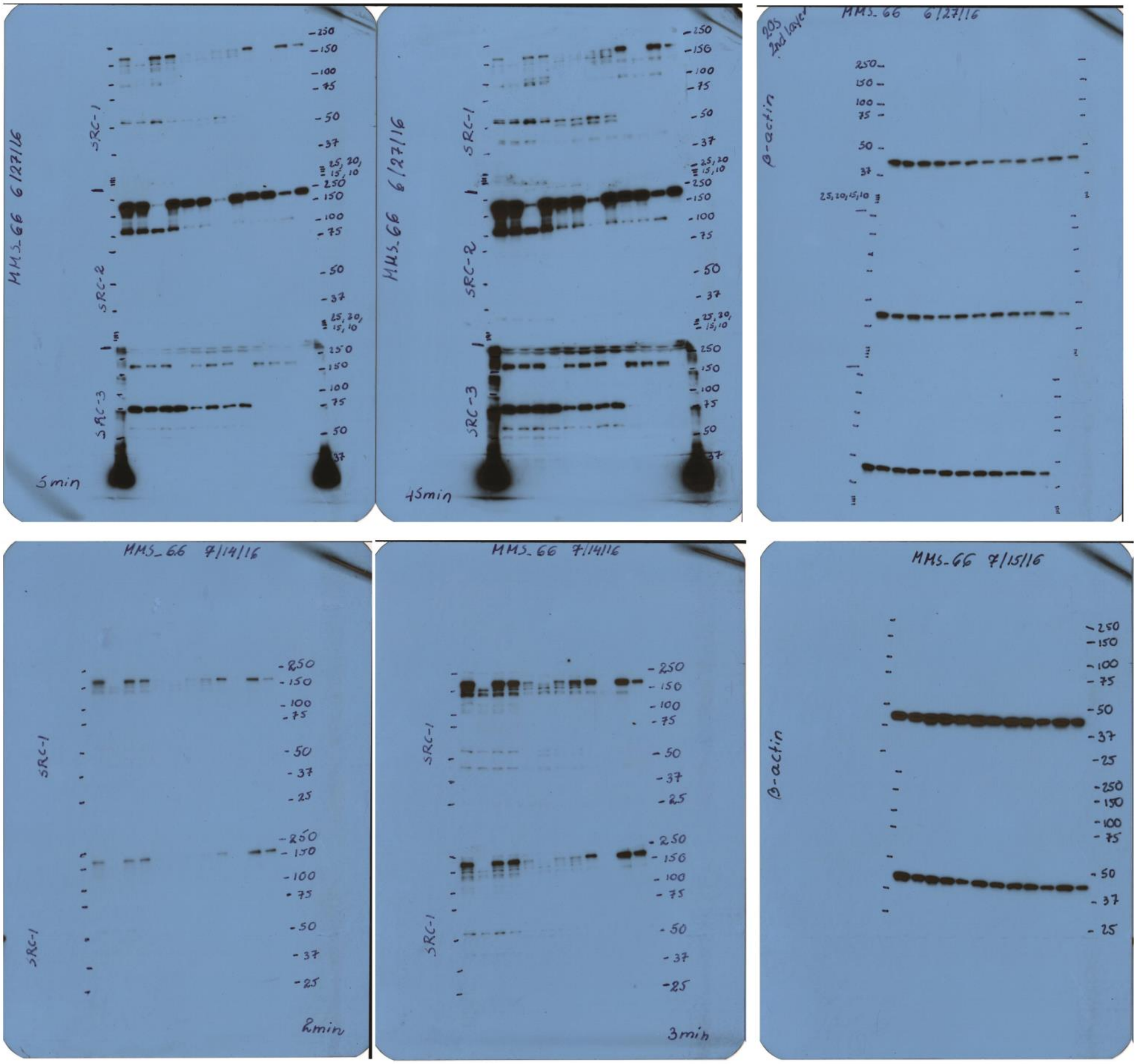

Supplemental Figure S1

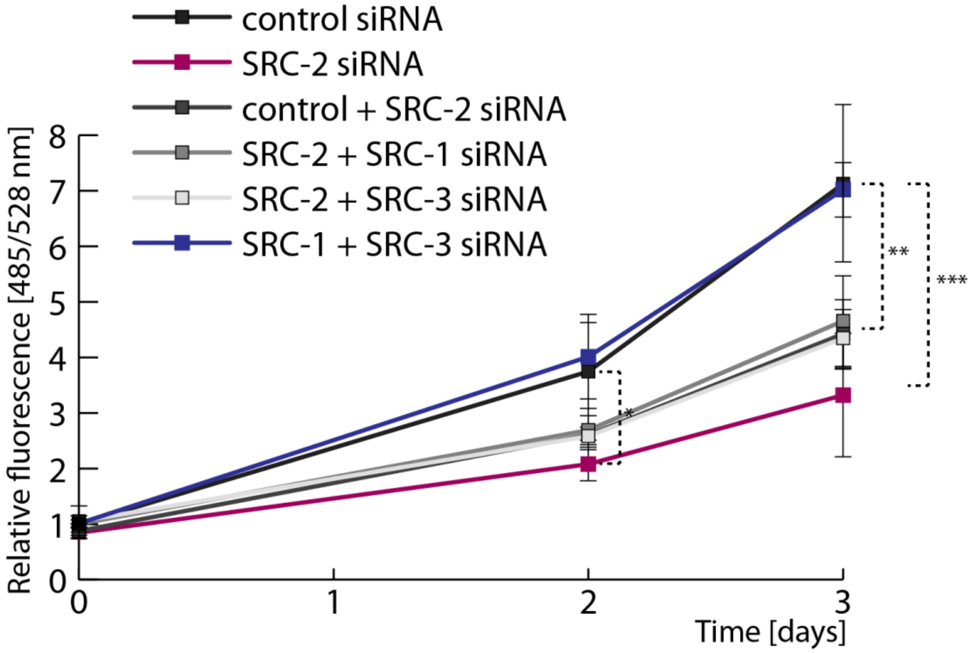

Supplemental Figure S2

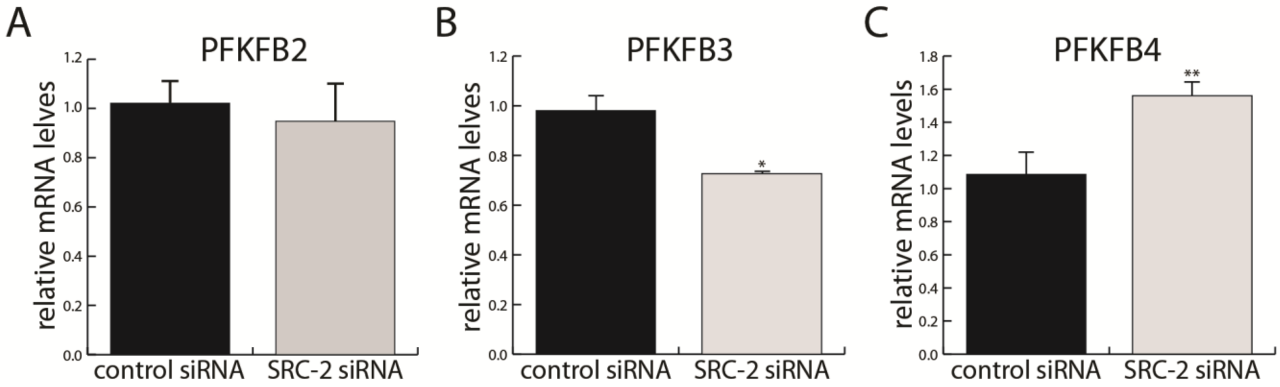

Supplemental Figure S3

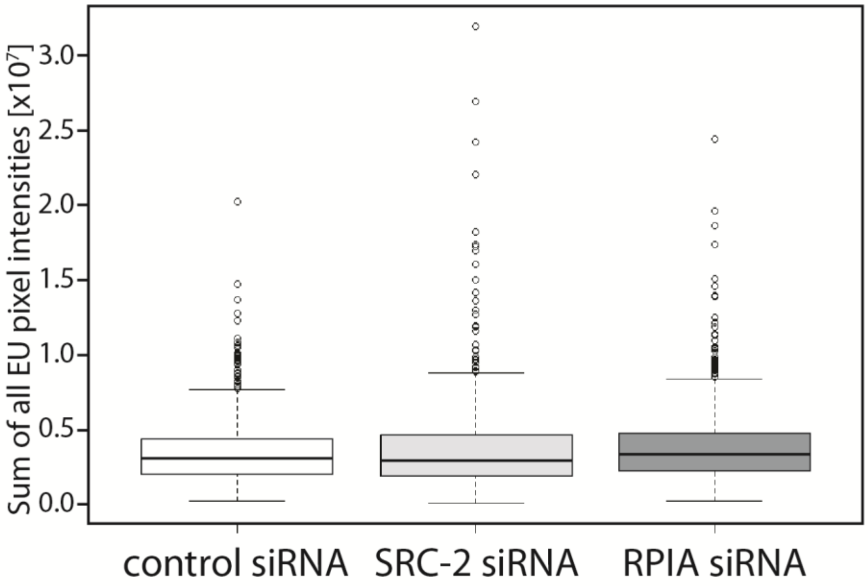

Supplemental Figure S4

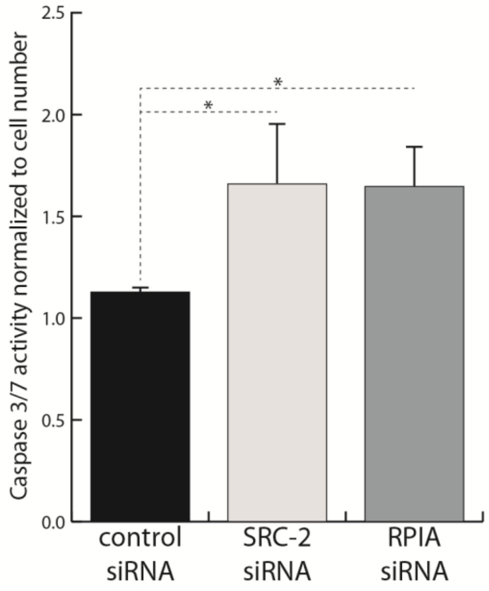

Supplemental Figure S5

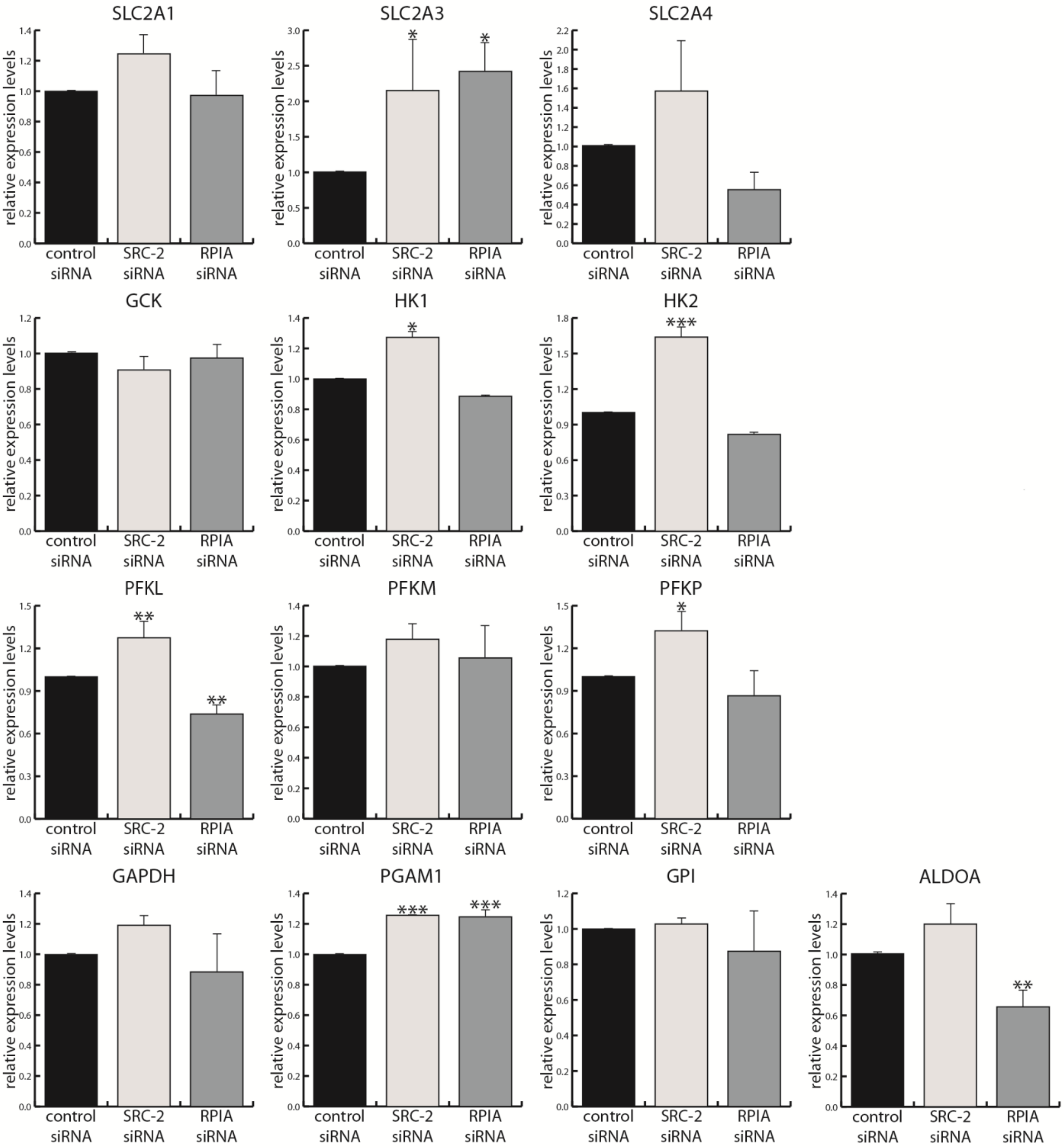

Supplemental Figure S6

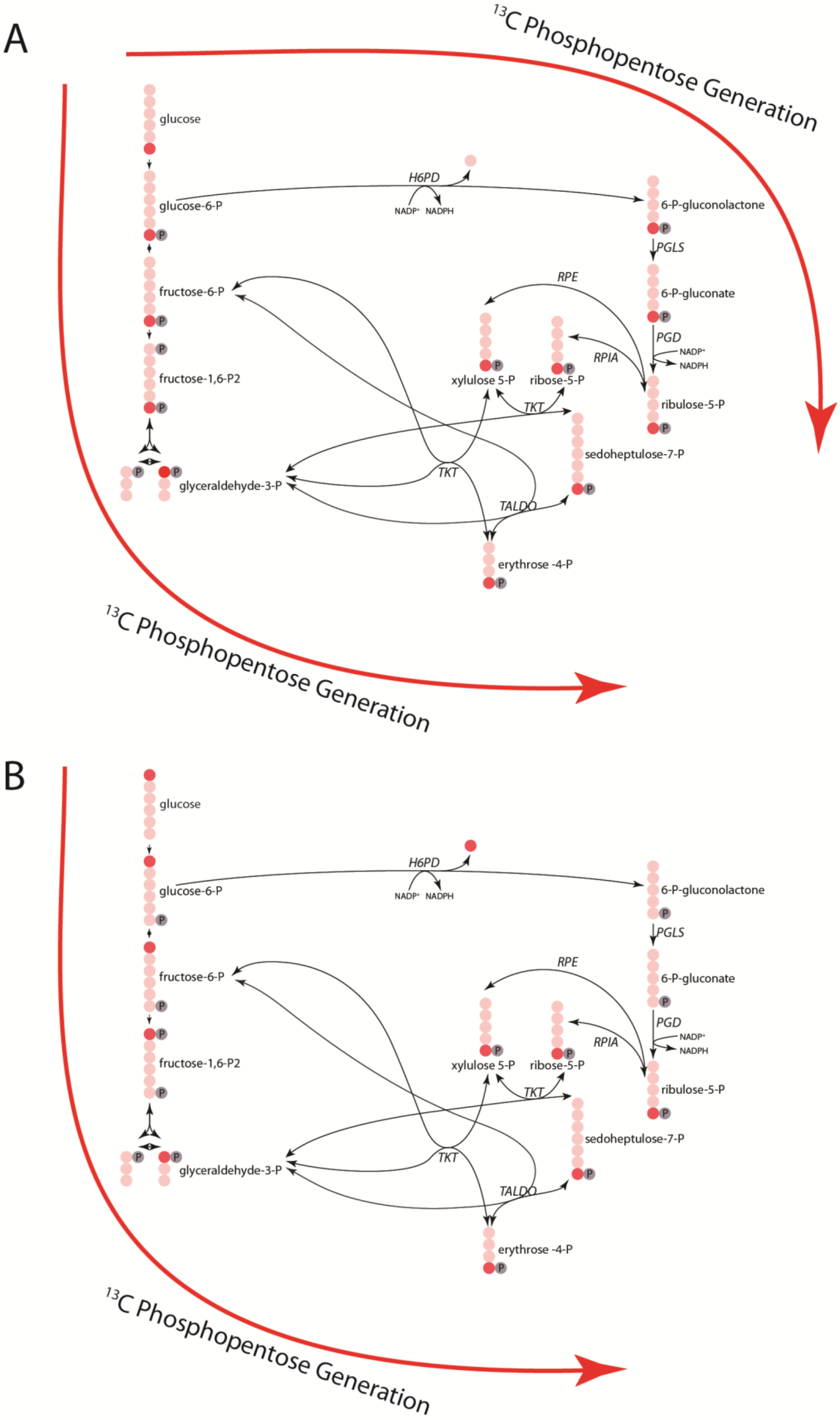

Supplemental Figure S7

	Applied Biosystems Taqman Gene Expression Assay
18S rRNA	4319413E
ALDOA	Hs00605108_g1
ALDOB	Hs01551887_m1
GAPDH	Hs02758991_g1
GCK	Hs01564555_m1
GPI	Hs00976715_m1
H6PD	Hs00188728_m1
HK1	Hs00175976_m1
HK2	Hs00606086_m1
PFKFB2	Hs01015408_m1
PFKFB3	Hs00998700_m1
PFKFB4	Hs00190096_m1
PFKL	Hs01040525_m1
PFKM	Hs00175997_m1
PFKP	Hs00242993_m1
PGAM1	Hs01652468_g1
PGD	Hs00427230_m1
PGLS	Hs00359986_m1
RPE	Hs00851816_g1
RPIA	Hs01107136_m1
SLC2A1	Hs00892681_m1
SLC2A3	Hs00359840_m1
SLC2A4	Hs00168966_m1
TALDO	Hs00997203_m1
TKT	Hs01115545_m1

Supplemental Table ST1
